# Diet-dependent acid load and type 2 diabetes: pooled results from three prospective cohort studies

**DOI:** 10.1007/s00125-016-4153-7

**Published:** 2016-11-17

**Authors:** Jessica C. Kiefte-de Jong, Yanping Li, Mu Chen, Gary C. Curhan, Josiemer Mattei, Vasanti S. Malik, John P. Forman, Oscar H. Franco, Frank B. Hu

**Affiliations:** 1 Department of Epidemiology, Erasmus MC, Room NA2903, PO Box 2040, 3000 CA Rotterdam, the Netherlands; 2 Department of Global Public Health, Leiden University College, The Hague, the Netherlands; 3 Department of Nutrition, Harvard T. H. Chan School of Public Health, Boston, MA USA; 4 Department of Epidemiology, Harvard T. H. Chan School of Public Health, Boston, MA USA; 5 Channing Division of Network Medicine, Department of Medicine, Brigham and Women’s Hospital, Harvard Medical School, Boston, MA USA

**Keywords:** Acid–base balance, Dietary acid load, Glucose intolerance, Insulin resistance

## Abstract

**Aims/hypothesis:**

Studies suggest a potential link between low-grade metabolic acidosis and type 2 diabetes. A western dietary pattern increases daily acid load but the association between diet-dependent acid load and type 2 diabetes is still unclear. This study aimed to assess whether diet-dependent acid load is associated with the risk of type 2 diabetes.

**Methods:**

We examined the association between energy-adjusted net endogenous acid production (NEAP), potential renal acid load (PRAL) and animal protein-to-potassium ratio (A:P) on incident type 2 diabetes in 67,433 women from the Nurses’ Health Study, 84,310 women from the Nurses’ Health Study II and 35,743 men from the Health Professionals’ Follow-up Study who were free from type 2 diabetes, cardiovascular disease and cancer at baseline. Study-specific HRs were estimated using Cox proportional hazards models with time-varying covariates and were pooled using a random effects meta-analysis.

**Results:**

We documented 15,305 cases of type 2 diabetes during 4,025,131 person-years of follow-up. After adjustment for diabetes risk factors, dietary NEAP, PRAL and A:P were positively associated with type 2 diabetes (pooled HR [95% CI] for highest (Q5) vs lowest quintile (Q1): 1.29 [1.22, 1.37], *p*_trend_ <0.0001; 1.29 [1.22, 1.36], *p*_trend_ <0.0001 and 1.32 [1.24, 1.40], *p*_trend_ <0.0001 for NEAP, PRAL and A:P, respectively). These results were not fully explained by other dietary factors including glycaemic load and dietary quality (HR [95% CI] for Q5 vs Q1: 1.21 [1.09, 1.33], *p*_trend_ <0.0001; 1.19 [1.08, 1.30] and 1.26 [1.17, 1.36], *p*_trend_ <0.0001 for NEAP, PRAL and A:P, respectively).

**Conclusions/interpretation:**

This study suggests that higher diet-dependent acid load is associated with an increased risk of type 2 diabetes. This association is not fully explained by diabetes risk factors and overall diet quality.

**Electronic supplementary material:**

The online version of this article (doi:10.1007/s00125-016-4153-7) contains peer-reviewed but unedited supplementary material, which is available to authorised users.

## Introduction

Type 2 diabetes is an important cause of mortality and morbidity globally and its increasing prevalence is driven by the obesity epidemic and ageing populations [1]. A western dietary pattern, characterised by high intake of foods containing animal products and beverages containing sugar and low intake of fruit, vegetables and whole grains, is associated with an increased risk of type 2 diabetes [2]. This association can be partially explained by low intake of whole grains and poor dietary fat quality, which have been related to glucose homeostasis in both experimental [3, 4] and observational studies [1, 2]. However, potential mechanisms behind other components of a western dietary pattern, such as foods rich in animal protein, are not yet fully elucidated.

It has been hypothesised that a western-style diet may cause low-grade metabolic acidosis, possibly leading to metabolic disturbances [5, 6]. Sulphur-containing amino acids (e.g. methionine and cysteine) are found in animal proteins, particularly in meat and fish, and are important determinants of dietary acid load due to sulfate generation after their oxidation [5]. Indeed, several studies have shown that the positive association between protein intake and type 2 diabetes is mainly driven by animal protein intake [7, 8].

Physiological effects of high dietary acid load include increased urinary excretion of sulfate, phosphorus and chloride, increased elimination of calcium, intrarenal vasodilatation and increased glomerular filtration rate [5, 9]. Hence, dietary acid load has been studied extensively in relation to kidney disease [10, 11], blood pressure [12, 13] and bone health [14]. Dietary acid load may also play a role in glucose homeostasis. Associations between markers of metabolic acidosis (i.e. low serum bicarbonate, higher anion gap and low urine pH) and insulin resistance have been reported in individuals with insulin resistance [15] and in a nested case–control study in type 2 diabetes [16]. In addition, a recent study found that dietary acid load was associated with insulin resistance in healthy Japanese workers [17]. The E3N-EPIC cohort study found diet-dependent acid load to be associated with an increased risk of type 2 diabetes [18]. However, this was not confirmed in a cohort of community-dwelling men in Sweden [19] and a Japanese cohort study only found an association between dietary acid load and type 2 diabetes risk in younger men [20]. These contrasting findings may be explained by differences in other dietary habits, sex, age and other population characteristics. Therefore, further research on this topic is needed to replicate and validate previous findings and facilitate dietary recommendations related to diet-dependent acid load for public health purposes. We aimed to evaluate the prospective association between dietary acid load and the incidence of type 2 diabetes in three cohorts of adults in the USA.

## Methods

### Study population

Data was used from three prospective cohort studies of health professionals in the USA: the Nurses’ Health Study (NHS), Nurses’ Health Study II (NHS2) and the Health Professionals’ Follow-up Study (HPFS).

The NHS was established in 1976 and included 121,700 registered nurses aged 30–55 years living in the 11 most populous states of the USA (California, Connecticut, Florida, Maryland, Massachusetts, Michigan, New Jersey, New York, Ohio, Pennsylvania and Texas). NHS2 was established in 1989 and included a younger population of nurses. The NHS2 population included 116,430 women between the ages of 25 and 42 years. The HPFS began in 1986 and included 51,529 men in health professions aged 40–75 years (29,683 dentists, 4185 pharmacists, 3745 optometrists, 2220 osteopath physicians, 1600 podiatrists and 10,098 veterinarians).

At baseline and every 2 years, participants in the NHS, NHS2 and HPFS completed questionnaires about diseases and health-related topics such as smoking, physical activity and medication use. In addition, food frequency questionnaires (FFQs) to assess dietary intake were administered at 4 year intervals. The follow-up rate was greater than 90% in all cohorts.

For the present analyses we excluded participants with prior diagnosis at baseline of cardiovascular disease (including coronary heart disease and stroke), cancer, type 1 or type 2 diabetes or history of gestational diabetes (in NHS cohorts only). Additionally, we excluded participants with implausible daily intakes of total energy (<2510 kJ or >14,644 kJ for women and <3347 kJ or >17,573 kJ for men) and participants with missing FFQ data. The final population of analyses consisted of 67,433 women in the NHS, 84,310 women in the NHS2 and 35,743 men in the HPFS.

The study protocols were approved by the institutional review board of Brigham and Women’s Hospital and Harvard T. H. Chan School of Public Health and all participants provided informed consent.

### Dietary assessment and dietary acid load

Habitual dietary intake over the preceding year was assessed by validated FFQs described in detail previously [21, 22]. Dietary data were collected in 1984 for the NHS, 1986 for the HPFS and 1991 for the NHS2 and were updated every 4 years with similar FFQs.

For each food item, participants were asked how often on average they consumed a serving of that food. The frequency of a food item was recorded as number of times per day, week or month.

To calculate nutrient intake, the frequency of consumption of each food item was multiplied by the nutrient content of one serving and then summed across all food items. Nutrient values of foods were obtained from the US Department of Agriculture [23].

We calculated dietary acid load by using three previously defined algorithms: net endogenous acid production (NEAP) [24], potential renal acid load (PRAL) [25] and animal protein-to-potassium ratio (A:P) [26]:

$$ \mathrm{NEAP}\kern0.15em \left(\mathrm{mEq}/\mathrm{day}\right)=\left(54.5\times \mathrm{protein}\left[\mathrm{g}/\mathrm{day}\right]/\mathrm{potassium}\kern0.15em \left[\mathrm{mEq}/\mathrm{day}\right]\right)-10.2 $$

$$ \begin{array}{l}\mathrm{PRAL}\kern0.2em \left(\mathrm{mEq}/\mathrm{day}\right)=0.4888\times \mathrm{protein}\ \left[\mathrm{g}/\mathrm{day}\right]+0.0366\times \mathrm{phosphorus}\ \left[\mathrm{mg}/\mathrm{day}\right]\hbox{--} 0.0205\times \hfill \\ {}\mathrm{potassium}\ \left[\mathrm{mg}/\mathrm{day}\right]\hbox{--} 0.0125\times \mathrm{calcium}\kern0.15em \left[\mathrm{mg}/\mathrm{day}\right]\hbox{--} 0.0263\times \mathrm{magnesium}\ \left[\mathrm{mg}/\mathrm{day}\right]\hfill \end{array} $$

$$ \mathrm{A}:\mathrm{P}=\mathrm{animal}\ \mathrm{protein}\kern0.2em \left(\mathrm{g}/\mathrm{day}\right)/\mathrm{potassium}\ \left(\mathrm{g}/\mathrm{day}\right) $$

The NEAP algorithm by Frassetto et al [24] estimates dietary acid load from an acid precursor (dietary protein) and an index of base precursors from organic anions (potassium) and has been previously validated in healthy men and women aged 17–73 years [24]. NEAP only includes protein and potassium as pH-altering nutrients from diet whereas the PRAL algorithm by Remer and Manz [25] estimates dietary acid load taking into account average intestinal absorption rates of ingested protein and additional minerals and has been validated against urine pH in healthy adults [25]. Since it is considered that animal protein constitutes a major determinant of dietary acid load [27], and NEAP and PRAL do not make a distinction between animal and vegetable protein, we also assessed the ratio of animal protein to potassium, as described previously [26].

To account for measurement error and to remove extraneous variation arising from total energy intake, all nutrients (including those for NEAP, PRAL and A:P) were adjusted for total energy intake by using the residual method [28].

The FFQs were validated against the diet records of 173 participants in the NHS in 1980 and 127 participants in the HPFS in 1986 [21, 22]. Correlation coefficients adjusted for energy and within-person variation varied from 0.52 (NHS) to 0.44 (HPFS) for total protein, from 0.53 (NHS) to 0.73 (HPFS) for potassium, from 0.51 (NHS) to 0.54 (HPFS) for calcium, from 0.53 (NHS) to 0.57 (HPFS) for phosphorus and 0.72 for magnesium when compared with 1 week food records [21, 22].

### Assessment of type 2 diabetes

Every 2 years, participants in all three cohorts were asked whether they had any physician diagnosis of diabetes. Participants who reported physician-diagnosed diabetes were sent a supplementary questionnaire to obtain information about symptoms, diagnostic tests and diabetes drug use.

Participants were originally defined as having type 2 diabetes if they experienced one or more symptoms of polydipsia, polyuria, weight loss and hunger and met at least one of the following criteria [29]: increased blood glucose levels (fasting levels ≥7.8 mmol/l, random blood levels ≥11.1 mmol/l and/or 2 h blood glucose levels ≥11.1 mmol/l during OGTT); raised blood glucose levels on two different occasions in the absence of symptoms; or treatment with glucose-lowering drugs.

In June 1998 the diagnostic criteria of type 2 diabetes were changed and a fasting blood glucose level of 7 mmol/l, rather than 7.8 mmol/l, was used as the threshold for diagnosis [30]. The questionnaires for the diagnosis of type 2 diabetes have been previously validated in subsamples of the cohorts [31, 32]. These validation studies showed that 98% and 97% of the cases of self-reported type 2 diabetes were confirmed by medical records in the NHS [32] and the HPFS, respectively [31, 32].

### Covariates

Updated information on anthropometric and lifestyle factors for type 2 diabetes, including body weight (height was ascertained at baseline), cigarette smoking, physical activity, family history of diabetes and history of kidney stones was collected in the 2-yearly questionnaires. Among participants in the NHS and NHS2, menopausal status and postmenopausal hormone use was ascertained by questionnaires; oral contraceptive use was ascertained by questionnaire in the NHS2 only. An approximation of moderate/vigorous physical activity levels was determined by multiplying the metabolic equivalent tasks (METs) measured in hours per week of each activity by the number of hours spent on the activity and taking the sum of these values (six METs or greater was defined as moderate/vigorous activity).

Based on the FFQ, an adherence score for the Alternative Healthy Eating Index (AHEI), an indicator of adherence to healthy eating behaviour, was derived as described in detail elsewhere [33], as well as indices of glycaemic load. Briefly, the AHEI included intake of the following foods and nutrients: vegetables, fruits, whole grains, sugar-sweetened beverages and fruit juice, nuts and legumes, red and processed meat, *trans* fat, long-chain *n*-3 fat, polyunsaturated fat and sodium. Alcohol intake was excluded from the AHEI and treated as an individual covariate. Each component was scored on a scale of 0–10 and the overall score ranged from 0–100, with a higher score representing a healthier diet. Principal component analysis was used for a posteriori-derived western-like dietary pattern scores as described in detail previously [34]. The overall glycaemic load was calculated by taking the sum of the following product: carbohydrates per food item × glycaemic index of food item × mean servings of food item per day [35].

### Statistical analyses

To assess long-term dietary acid load exposure and to reduce within-person variation, we calculated the cumulative average of dietary acid load from baseline until type 2 diabetes diagnosis, death or end of follow-up, whichever came first. Missing dietary data on follow-up visits were replaced by the cumulative average of prior dietary assessments.

Participant characteristics by dietary acid load were standardised to the age distribution of the study population.

We used multivariable Cox proportional hazard models with time-dependent covariates to assess the relationship between quintiles of NEAP, PRAL and A:P and type 2 diabetes. Linear trends across the quintiles were examined by using the median values of each quintile of dietary acid load as continuous variable in the analyses.

To account for differences in age distribution, time-dependent Cox regression analyses were stratified by age in months (i.e. an age-adjusted model allowing for different baseline hazard functions for different age groups).

Results were further adjusted for total energy intake (quintiles), BMI (continuously), family history of type 2 diabetes (yes/no), menopausal status (premenopausal, postmenopausal without hormone use, postmenopausal with past hormone use, postmenopausal with current hormone use), history of hypertension and hypercholesterolaemia (yes/no), smoking status (never smoking, former smoker, current smoker [1–14 cigarettes, 15–24 cigarettes and ≥25 cigarettes/day]), alcohol intake (0, 0.1–4.9, 5.0–14.9, 15.0–19.9, 20.0–29.9 and ≥30 g/day) and moderate/vigorous physical activity (0, 0.01–1.0, 1.0–3.5, 3.5–6.0 and ≥6.0 h/week) (multivariate model 1). Additionally we adjusted the analyses for other dietary exposures associated with type 2 diabetes [1, 2] such as glycaemic load (quintiles), AHEI (quintiles) and the western dietary pattern (quintiles) (multivariate model 2).

Sensitivity analyses were performed by stratification according to BMI status (<25 kg/m^2^, 25–29 kg/m^2^ and ≥30 kg/m^2^), age (<60 years and ≥60 years), smoking status (ever vs never smoker), presence of hypertension and history of kidney stones [34]. Effect modification by BMI, smoking status, hypertension and kidney stones was evaluated by using the likelihood ratio test. Furthermore, since it has recently been found that animal protein but not vegetable protein was associated with an increased risk of type 2 diabetes [8], we additionally adjusted the association between NEAP and type 2 diabetes for animal protein intake (quintiles). Last, to assess the potential influence of dietary changes due to diabetes-related symptoms before diagnosis, we used a 4 year lag between dietary acid load and type 2 diabetes in sensitivity analysis. Results from the three cohorts were pooled using random effects meta-analysis and are reported as HR and 95% CI. All analyses were performed with SAS 9.3 Unix (SAS Institute, Cary, NC, USA). A *p* value <0.05 was considered as statistically significant.

## Results

### Characteristics of the study population

Mean (SD) dietary NEAP, PRAL and A:P was, respectively, 43.8 (8.7) mEq/day, −3.1 (10.6) mEq/day and 18.4 (4.1) for the NHS, 49.9 (10.5) mEq/day, 6.4 (12.2) mEq/day and 20.3 (5.1) for the NHS2 and 47.7 (10.5) mEq/day, 5.7 (13.2) mEq/day and 19.5 (5.1) for the HPFS.

Participants with high dietary NEAP tended to consume more red meat and fish and to consume fruit, whole grains and sugar-containing beverages less often. In addition, participants with high dietary NEAP were likely to consume less alcohol and had an overall lower AHEI score and a higher western dietary pattern score (Table 1).

**Table 1:** Age-adjusted characteristics of the NHS and HPFS at median follow-up time

Characteristic	NHS (1994)^a^		NHS2 (1999)^a^		HPFS (1996)^a^	
	Quintile 1 (*n* = 11,449)	Quintile 5 (*n* = 14,974)	Quintile 1 (*n* = 18,030)	Quintile 5 (*n* = 13,878)	Quintile 1 (*n* = 6428)	Quintile 5 (*n* = 7472)
Age, years^b^	62.4 (6.8)	57.6 (6.8)	45.1 (4.5)	43.4 (4.7)	64.6 (9.4)	59.5 (8.4)
Premenopausal, %	10.2	11.0	74.1	70.5	−	−
Family history of diabetes, %	17.3	19.1	14.9	17.4	19.1	20.2
BMI, kg/m^2^	24.9 (4.4)	27.3 (5.6)	24.8 (5.0)	28.0 (6.9)	25.4 (3.5)	26.5 (3.9)
Hypertension, %	21.3	24.4	9.3	15.2	35.6	36.6
Hypercholesterolaemia, %	27.7	30.1	18.6	24.6	41.0	43.4
Moderate/vigorous intensity activity, h/week	2.6 (4.1)	1.5 (3.0)	3.0 (4.0)	1.7 (2.8)	5.0 (6.4)	3.1 (5.0)
Current smoker, %	14.2	13.0	10.1	8.5	4.4	7.1
Alcohol intake, g/day	5.8 (10.1)	4.5 (9.1)	5.0 (8.3)	2.7 (6.4)	11.8 (15.8)	9.7 (14.0)
AHEI score	50.6 (10.1)	45.0 (9.8)	50.1 (9.5)	41.3 (8.5)	53.0 (9.0)	44.0 (9.3)
Western dietary pattern score	0.4 (0.9)	0.6 (0.8)	−0.3 (0.7)	0.1 (0.8)	−0.4 (0.7)	0.2 (1.0)
Glycaemic load, units/day	113.5 (16.8)	95.6 (15.7)	134.5 (18.6)	114.2 (19.5)	145.6 (23.9)	117.5 (22.3)
Red meat intake, no. of servings/day	0.8 (0.5)	1.1 (0.6)	0.6 (0.4)	1.2 (0.6)	0.7 (0.5)	1.4 (0.9)
Sugar-containing beverage intake, no. of servings/day	1.5 (0.9)	0.9 (0.6)	1.4 (1.2)	0.9 (1.0)	1.5 (1.2)	1.0 (0.8)
Dairy intake, no. of servings/day	1.6 (0.9)	1.4 (0.8)	2.3 (1.3)	1.8 (1.1)	1.9 (1.3)	1.7 (1.2)
Vegetable intake, no. of servings/day	4.3 (1.7)	3.4 (1.2)	3.9 (2.0)	2.3 (1.1)	3.9 (2.0)	2.4 (1.2)
Nut intake, no. of servings/day	1.2 (0.8)	0.8 (0.6)	0.4 (0.4)	0.2 (0.2)	0.6 (0.5)	0.4 (0.4)
Fruit intake, no. of servings/day	1.7 (1.0)	0.8 (0.5)	1.8 (1.1)	0.7 (0.5)	2.6 (1.6)	1.0 (0.7)
Whole grain intake, g/day	15.4 (10.4)	11.1 (7.9)	28.3 (15.9)	18.2 (11.8)	32.3 (20.1)	20.3 (15.5)
Fish intake, no. of servings/day	0.2 (0.2)	0.3 (0.2)	0.2 (0.2)	0.2 (0.2)	0.3 (0.3)	0.4 (0.3)
Energy intake, kJ/day	7196 (2192)	6987 (2205)	7510 (2025)	7234 (2058)	8284 (2251)	2029 (1017)
Fat intake, % of energy	29.0 (4.9)	34.7 (4.6)	27.5 (5.0)	34.0 (5.1)	26.7 (5.5)	34.2 (5.3)
Saturated fat intake, % of energy	10.1 (2.2)	12.2 (2.1)	9.3 (2.1)	11.9 (2.2)	8.6 (2.3)	11.7 (2.4)
Polyunsaturated fat intake, % of energy	4.5 (1.3)	4.9 (1.2)	5.1 (1.2)	5.7 (1.2)	5.3 (1.4)	5.9 (1.2)
Carbohydrate intake, % of energy	54.5 (6.6)	44.8 (6.1)	56.4 (6.3)	45.8 (6.4)	55.6 (7.6)	43.5 (6.8)
Protein intake,% of energy	16.6 (2.3)	19.8 (2.9)	17.0 (2.5)	20.4 (3.1)	16.3 (2.4)	20.0 (3.1)
Animal protein intake, % of energy	11.1 (2.5)	15.1 (2.9)	11.0 (2.8)	15.7 (3.0)	10.4 (2.6)	15.3 (3.1)
Potassium intake, mg/day	3583 (930)	2673 (688)	3449 (941)	2449 (708)	3875 (1060)	2805 (802)
Dietary NEAP, mEq/day	32.1 (3.5)	55.7 (6.0)	36.8 (4.5)	66.7 (7.3)	33.8 (4.4)	62.5 (7.4)
Dietary PRAL, mEq/day	−17.4 (8.0)	8.7 (6.4)	−8.8 (9.1)	22.0 (7.3)	−12.9 (10.3)	21.3 (7.2)
Dietary A:P	13.2 (2.2)	23.5 (3.0)	14.3 (3.0)	27.8 (3.8)	13.2 (2.8)	26.1 (3.9)

Values are expressed as means (SD) or %, standardised to the age distribution of the study population and grouped according to quintiles of NEAP (quintile 1, low; quintile 5, high)

^a^Year represents median follow-up

^b^Value is not age-adjusted

During 1,709,638 person-years of follow-up, 7655 new cases with type 2 diabetes were ascertained in the NHS; during 1,513,932 person-years of follow-up, 4109 cases were ascertained in the NHS2 and during 801,561 person-years of follow-up 3541 cases were ascertained in HPFS.

### Dietary acid load and type 2 diabetes

The association between indices of dietary acid load and type 2 diabetes is shown in Table 2. In the NHS and HPFS cohorts, dietary NEAP was associated with an increased risk of type 2 diabetes after adjustment for diabetes risk factors (HR [95% CI] for highest vs lowest quintile of NEAP was 1.28 [1.18, 1.38], *p*_trend_ <0.0001 for NHS, 1.30 [1.17, 1.44], *p*_trend_ <0.0001 for NHS2 and 1.32 [1.18, 1.47], *p*_trend_ <0.0001 for HPFS).

**Table 2:** Association of type 2 diabetes mellitus with NEAP, PRAL and A:P

Model^a^	Quintile 1	Quintile 2	Quintile 3	Quintile 4	Quintile 5	*p*_trend_
NHS						
Dietary NEAP						
No. of cases/person-years	1051/342,428	1373/341,989	1598/341,556	1699/341,826	1934/341,838	
Age-adjusted	1.00	1.27 (1.18, 1.38)*	1.50 (1.39, 1.62)*	1.65 (1.53, 1.79)*	2.07 (1.91, 2.23)*	<0.0001
Multivariate model 1	1.00	1.13 (1.05, 1.23)*	1.23 (1.13, 1.33)*	1.22 (1.12, 1.32)*	1.28 (1.18, 1.38)*	<0.0001
Multivariate model 2	1.00	1.14 (1.05, 1.23)*	1.23 (1.13, 1.34)*	1.23 (1.13, 1.33)*	1.29 (1.19, 1.41)*	<0.0001
Dietary PRAL						
No. of cases/person-years	1083/342,058	1382/341,855	1516/341,733	1708/341,945	1966/342,047	
Age-adjusted	1.00	1.27 (1.17, 1.37)*	1.42 (1.31, 1.54)*	1.67 (1.31, 1.54)*	2.15 (1.99, 2.32)*	<0.0001
Multivariate model 1	1.00	1.13 (1.04, 1.22)*	1.17 (1.08, 1.27)*	1.21 (1.12, 1.31)*	1.26 (1.16, 1.36)*	<0.0001
Multivariate model 2	1.00	1.10 (1.02, 1.20)*	1.14 (1.05, 1.24)*	1.18 (1.09, 1.28)*	1.23 (1.13, 1.33)*	<0.0001
Dietary A:P						
No. of cases/person-years	1062/342,228	1332/341,912	1611/341,662	1738/341,775	1912/342,026	
Age-adjusted	1.00	1.23 (1.14, 1.34)*	1.51 (1.40, 1.63)*	1.70 (1.57, 1.83)*	2.08 (1.93, 2.25)*	<0.0001
Multivariate model 1	1.00	1.10 (1.01, 1.19)*	1.21 (1.12, 1.31)*	1.22 (1.13, 1.32)*	1.26 (1.16, 1.36)*	<0.0001
Multivariate model 2	1.00	1.11 (1.02, 1.20)*	1.23 (1.13, 1.34)*	1.26 (1.15, 1.37)*	1.31 (1.19, 1.43)*	<0.0001
NHS2						
Dietary NEAP						
No. of cases/person-years	595/303,044	752/302,999	819/302,865	904/302,564	1039//302,459	
Age-adjusted	1.00	1.32 (1.19, 1.47)*	1.57 (1.41, 1.75)*	1.98 (1.78, 2.19)*	2.82 (2.54, 3.12)*	<0.0001
Multivariate model 1	1.00	1.03 (0.93, 1.15)	1.07 (0.96, 1.19)	1.14 (1.03, 1.27)*	1.30 (1.17, 1.44)*	<0.0001
Multivariate model 2	1.00	1.01 (0.91, 1.13)	1.04 (0.93, 1.16)	1.10 (0.98, 1.23)	1.22 (1.09, 1.37)*	<0.0001
Dietary PRAL						
No. of cases/person-years	684/303,268	806/303,105	820/302,908	861/302,517	952/302,395	
Age-adjusted	1.00	1.34 (1.21, 1.48)*	1.57 (1.42, 1.74)*	1.95 (1.76, 2.16)*	2.83 (2.55, 3.13)*	<0.0001
Multivariate model 1	1.00	1.13 (1.02, 1.25)*	1.11 (1.00, 1.23)*	1.17 (1.05, 1.30)*	1.33 (1.20, 1.48)*	<0.0001
Multivariate model 2	1.00	1.10 (0.99, 1.22)	1.07 (0.96, 1.19)	1.30 (1.00, 1.24)*	1.25 (1.12, 1.40)*	0.0065
Dietary A:P						
No. of cases/person-years	563/302,924	749/303,056	839/302,969	896/302,618	1026/302,366	
Age-adjusted	1.00	1.37 (1.22, 1.52)*	1.69 (1.51, 1.88)*	2.06 (1.85, 2.29)*	3.07 (2.77, 3.41)*	<0.0001
Multivariate model 1	1.00	1.08 (0.97, 1.21)	1.09 (0.97, 1.21)	1.18 (1.06, 1.32)*	1.35 (1.21, 1.50)*	<0.0001
Multivariate model 2	1.00	1.07 (0.95, 1.20)	1.06 (0.95, 1.19)	1.15 (1.02, 1.29)*	1.30 (1.15, 1.47)*	<0.0001
HPFS						
Dietary NEAP						
No. of cases/person-years	585/160,351	636/160,660	701/160,617	770/160,289	849/159,654	
Age-adjusted	1.00	1.07 (0.95, 1.20)	1.21 (1.08, 1.35)*	1.41 (1.27, 1.57)*	1.74 (1.56, 1.94)*	<0.0001
Multivariate model 1	1.00	1.00 (0.89, 1.12)	1.08 (0.96, 1.20)	1.16 (1.04, 1.29)*	1.32 (1.18, 1.47)*	<0.0001
Multivariate model 2	1.00	0.92 (0.82, 1.03)	0.95 (0.84, 1.06)	0.99 (0.88, 1.11)	1.09 (0.96, 1.23)	0. 0370
Dietary PRAL						
No. of cases/person-years	585/160,199	688/160,651	694/160,536	752/160,402	825/159,773	
Age-adjusted	1.00	1.17 (1.04, 1.30)*	1.21 (1.08, 1.35)*	1.39 (1.25, 1.55)*	1.71 (1.54, 1.91)*	<0.0001
Multivariate model 1	1.00	1.10 (0.98, 1.23)	1.09 (0.97, 1.22)	1.17 (1.04, 1.30)*	1.29 (1.16, 1.44)*	<0.0001
Multivariate model 2	1.00	1.01 (0.90, 1.13)	0.95 (0.85, 1.07)	0.99 (0.88, 1.12)	1.07 (0.94, 1.20)	0.3583
Dietary A:P						
No. of cases/person-years	553/160,474	633/160,629	662/160,617	816/160,334	877/159,507	
Age-adjusted	1.00	1.1 (1.02, 1.28)*	1.22 (1.09, 1.37)*	1.60 (1.44, 1.79)*	1.94 (1.74, 2.16)*	<0.0001
Multivariate model 1	1.00	1.04 (0.92, 1.16)	1.03 (0.92, 1.15)	1.26 (1.12, 1.40)*	1.39 (1.25, 1.55)*	<0.0001
Multivariate model 2	1.00	0.94 (0.84, 1.06)	0.90 (0.80, 1.01)	1.07 (0.94, 1.20)	1.15 (1.01, 1.31)*	0.0016
Pooled cohorts						
Dietary NEAP						
Age-adjusted	1.00	1.22 (1.09, 1.37)*	1.42 (1.23, 1.64)*	1.67 (1.40, 1.98)*	2.16 (1.68, 2.79)*	<0.0001
Multivariate model 1	1.00	1.06 (0.98, 1.15)	1.13 (1.03, 1.24)*	1.18 (1.11, 1.25)*	1.29 (1.22, 1.37)*	<0.0001
Multivariate model 2	1.00	1.03 (0.91, 1.16)	1.07 (0.91, 1.26)	1.10 (0.97, 1.26)	1.21 (1.09, 1.33)*	<0.0001
Dietary PRAL						
Age-adjusted	1.00	1.26 (1.17, 1.35)*	1.39 (1.22, 1.60)*	1.66 (1.39, 1.97)*	2.19 (1.64, 2.94)*	<0.0001
Multivariate model 1	1.00	1.12 (1.06, 1.18)*	1.13 (1.07, 1.20)*	1.19 (1.13, 1.26)*	1.29 (1.22, 1.36)*	<0.0001
Multivariate model 2	1.00	1.08 (1.02, 1.14)*	1.06 (0.96, 1.18)	1.10 (1.06, 1.22)*	1.19 (1.08, 1.30)*	0.0093
Dietary A:P						
Age-adjusted	1.00	1.24 (1.13, 1.36)*	1.46 (1.24, 1.72)*	1.77 (1.55, 2.04)*	2.31 (1.78, 3.02)*	<0.0001
Multivariate model 1	1.00	1.08 (1.02, 1.14)*	1.11 (1.00, 1.23)*	1.22 (1.15, 1.29)*	1.32 (1.24, 1.40)*	<0.0001
Multivariate model 2	1.00	1.04 (0.95, 1.15)	1.06 (0.88, 1.27)	1.16 (1.06, 1.28) ^a^	1.26 (1.17, 1.36)*	<0.0001

Data are shown as HR (95% CI). The results were pooled using random effect meta-analysis

^a^Multivariate model 1: additionally adjusted for total energy intake (quintiles), BMI (continuously), family history of diabetes (yes/no), menopausal status (premenopausal or postmenopausal, never, past or current menopausal hormone use), presence of hypertension and hypercholesterolaemia (yes/no), smoking status (never smoker, former smoker, current smoker: 1–14, 15–24 or ≥25 cigarettes/day), alcohol intake (0, 0.1–4.9, 5.0–14.9, 15.0–19.9, 20.0–29.9 and ≥30 g/day), moderate/vigorous intensity activity (0, 0.01–1.0, 1.0–3.5, 3.5–6.0 and ≥6 h/week). Multivariate model 2: additionally adjusted for glycaemic load (quintiles), AHEI index (quintiles; including the following components: sugar-containing beverages, fruit, vegetables, nuts, red meat, whole grains, EPA, DHA, other PUFAs, *trans* fat, and sodium) and the western dietary pattern (quintiles)

*p < 0.05

Similar results were found for PRAL. Higher PRAL was associated with an increased risk of type 2 diabetes after adjustment for diabetes risk factors (HR [95% CI] for highest vs lowest quintile was 1.26 [1.16, 1.36], *p*_trend_ <0.0001 for NHS, 1.33 [1.20, 1.48], *p*_trend_ <0.0001 for NHS2 and 1.29 [1.16, 1.44], *p*_trend_ <0.0001 for HPFS).

In addition, a higher A:P was associated with an increased risk of type 2 diabetes after adjustment for diabetes risk factors (HR [95% CI] for highest vs lowest quintile was 1.26 [1.16, 1.36], *p*_trend_ <0.0001 for NHS, 1.35 [1.21, 1.50], *p*_trend_ <0.0001 for NHS2 and 1.39 [1.25, 1.55], *p*_trend_ <0.0001 for HPFS). Additional adjustment for dietary risk factors for type 2 diabetes attenuated some of the associations but they remained statistically significant except for PRAL in HPFS (HR [95% CI] for highest vs lowest quintile of NEAP was 1.29 [1.19, 1.41], *p*_trend_ <0.0001 for NHS, 1.22 [1.09, 1.37], *p*_trend_ <0.0001 for NHS2 and 1.09 [0.96, 1.23], *p*_trend_ = 0.0370 for HPFS and HR [95% CI] for PRAL was 1.23 [1.13, 1.33], *p*_trend_ <0.0001 for NHS, 1.25 [1.12, 1.40], *p*_trend_ <0.0001 for NHS2 and 1.07 [0.94, 1.20], *p*_trend_ = 0.358 for HPFS; Table 2). The pooled estimate of HR (95% CI) for type 2 diabetes risk according to dietary NEAP, PRAL and A:P was 1.21 (1.09, 1.33), *p*_trend_ <0.0001, 1.19 (1.08, 1.30) *p*_trend_ = 0.009 and 1.26 (1.17, 1.36), *p*_trend_ <0.0001, respectively, for highest vs lowest quintile after adjustment for diabetes and dietary risk factors.

### Additional analyses

After stratification by BMI, the associations between type 2 diabetes and NEAP, PRAL and A:P were slightly stronger in participants with BMI <25 kg/m^2^ than in those who were overweight or obese but the difference did not reach statistical significance across strata (HR [95% CI] for highest vs lowest quintile of NEAP was 1.33 [1.13, 1.57] for participants with BMI <25 kg/m^2^, 1.24 [1.12, 1.38] for overweight participants and 1.14 [0.96, 1.35] for obese participants, *p*_interaction_ = 0.4237; electronic supplementary material [ESM] Table 1). Also, stratification by smoking status and hypertension yielded comparable results (HR [95% CI] for highest vs lowest quintile of NEAP was 1.23 [1.10, 1.39] for never smokers and 1.20 [1.08, 1.33] for ever smokers, *p*_interaction_ = 0.3272, and 1.25 [1.15, 1.36] for participants without hypertension and 1.17 [0.99, 1.38] for participants with hypertension, *p*_interaction_ = 0.8875; ESM Table 1). In addition, no differential associations were found after stratification by history of kidney stones and age (HR [95% CI] for highest vs lowest quintile of NEAP was 0.99 [0.70, 1.25] for those with kidney stones and 1.11 [0.99, 1.29] for those without kidney stones, *p*_interaction_ = 0.3089, and 1.19 [1.02, 1.38] for participants below 60 years of age and 1.13 [0.98, 1.31] for those aged 60 years and above; ESM Table 1).

Results did not differ in sensitivity analyses using a 4 year lag of exposure to dietary acid load (HR [95% CI] for highest vs lowest quintile of NEAP was 1.30 [1.18, 1.41], *p*_trend_ <0.0001 for NHS, 1.24 [1.10, 1.39], *p*_trend_ <0.0001 for NHS2 and 1.13 [0.99, 1.28], *p*_trend_ = 0.0026 for HPFS). Additional adjustment for animal protein weakened the association between dietary NEAP and type 2 diabetes but results remained significant among women in NHS and NHS2 (HR [95% CI] for highest vs lowest quintile was 1.23 [1.12, 1.34], *p*_trend_ <0.0001 for NHS, 1.14 [1.01, 1.29], *p*_trend_ = 0.0103 for NHS2 and 0.97 [0.85, 1.11], *p*_trend_ = 0.8328 for HPFS).

## Discussion

In three prospective cohorts of men and women in the USA, we found that dietary acid load was associated with an increased risk of type 2 diabetes. In addition, differences in BMI and other dietary risk factors for type 2 diabetes, such as glycaemic load, the AHEI index and an a priori-defined western dietary pattern, did not fully explain the observed associations.

### Comparison with other studies

The results of this study are in line with results from the E3N-EPIC cohort study wherein dietary acid load was found to be associated with the risk of type 2 diabetes in a cohort of French women aged ∼50 years [18]. Another study revealed that dietary NEAP and PRAL were associated with insulin resistance and beta cell function in Japanese men and women aged 19–69 years, with the latter association being mainly present in normal-weight individuals [17]. Recent findings from The Japan Public Health Center-based Prospective Study showed that PRAL but not NEAP was associated with the risk of type 2 diabetes only in Japanese men [20]. Moreover, the E3N-EPIC study found that the association between dietary acid load and type 2 diabetes was stronger in women with BMI <25 kg/m^2^, which is in line with our findings. In contrast, another study in community-dwelling older men (aged 70–71 years) did not confirm the aforementioned associations [19]. It has been proposed that these contrasting results may be due to differences in age distribution since the results from Akter et al showed that the association between dietary acid load and type 2 diabetes was mainly observed in younger individuals [20]. However, our stratified analyses did not reveal any significant effect modification by age on the association between dietary acid load and type 2 diabetes. We found that the association between diet acid load and diabetes risk was slightly stronger in women from the NHS than in men from the HPFS, suggesting that diet-dependent acid load may affect the development of type 2 diabetes in a sex-specific manner (e.g. due to differences in sex hormones, such as oestrogen, which may affect acid–base balance) [36].

### Potential mechanisms

Dietary acid load was determined by validated indices based on protein and potassium intake. We have previously shown that animal protein but not vegetable protein is associated with increased type 2 diabetes [8]. In this analysis, we found that the association between dietary NEAP and type 2 diabetes was explained by animal protein intake to some extent. Sulphur-containing amino acids, such as methionine and cysteine, are found in animal protein and are main determinants of acid load as sulfate is generated after their oxidation [37]. Some studies of animal protein in relation to type 2 diabetes have shown conflicting results [38]. Nevertheless, red meat intake, a major source of sulphur-containing amino acids, has been consistently associated with an increased risk of insulin resistance, the metabolic syndrome and type 2 diabetes [39].

The main food sources of potassium are fruit and vegetables, which also provide other base cations (e.g. magnesium) [23]. A previous review showed that green leafy vegetable intake was associated with a reduced risk of type 2 diabetes but inconsistent results were found for intake of other vegetables and fruit [40]. Potassium is involved in acid–base balance by assisting electro-neutrality through exchange across cellular membranes for hydrogen ions [41, 42]. As a result, potassium-rich foods have been shown to be more alkalising than animal product-based foods [43].

The hypothesis that low-grade-metabolic acidosis may play a role in the aetiology of type 2 diabetes has been suggested by others. Some experimental studies have shown that reduction of the extracellular pH decreases beta cell response [44], reduces insulin secretion [44] and increases cortisol production [45], which in turn may affect the development of type 2 diabetes [46]. In addition, several studies have confirmed that markers of low-grade metabolic acidosis, such as lower plasma bicarbonate [6, 15, 16], higher anion gap [15], lower urine pH [47] and high levels of plasma lactate [48], are linked to insulin resistance, suggesting that low-grade metabolic acidosis could be involved in the aetiology of type 2 diabetes.

### Methodological considerations

Strengths of this study include the prospective design with long follow-up, the large sample size, high rates of follow-up and repeated dietary measurements during follow-up. In addition, the wide range of data on confounding variables and the homogeneity of the study population help to reduce residual confounding. However, several limitations need to be considered. First, dietary data was based on self-reported FFQs. While these are susceptible to measurement error, we used energy-adjusted nutrient intake for the calculation of dietary acid load as well as repeated measurements of diet, which can reduce the magnitude of measurement error [49]. Nonetheless, phosphorus intake for the calculation of PRAL did not include the assessment of phosphorus-containing food additives. Although the results for PRAL were similar to those for NEAP and A:P, they should be interpreted with caution. Second, we did not have data on kidney function available in the cohorts. Since kidney function is an important determinant of acid–base balance [27], it may be speculated that the relationship between dietary acid load and type 2 diabetes is more pronounced in participants with impaired kidney function due to altered haemodynamic adaptation to high acid load [10]. Hence, future studies on dietary acid load that include detailed urinary markers of acid–base balance (e.g. ammonium) and kidney function are needed to clarify the observed associations. Third, this study is of an observational nature. Although we adjusted for many confounders, residual confounding cannot be fully excluded. Last, the study population consisted primarily of white health professionals. This may limit the generalisability of our study results but could strengthen internal validity since confounding by ethnicity and socioeconomic status is greatly reduced.

### Public health implications

It has recently been demonstrated in healthy individuals that both the capillary and urine pH can be modified by a diet high or low in protein combined with a high or low intake of fruit and vegetables [43]. Therefore, our findings may have important public health implications for the future. If future studies confirm that food-induced improvement in acid–base balance is accompanied by improved insulin sensitivity, this may facilitate development of dietary guidelines, such as more specific recommendations on the ratio of potassium to protein in diets.

### Conclusions

Findings from this prospective study in men and women suggest that a higher dietary acid load is associated with an increased risk of type 2 diabetes. These results are not fully explained by BMI or diet-related risk factors associated with type 2 diabetes, such as glycaemic index and overall healthy or western dietary patterns. Randomised controlled trials are needed to clarify whether specific dietary interventions to reduce dietary acid load (e.g. a diet high in fruit and vegetables and low in animal protein) could improve glucose homeostasis and reduce the risk of type 2 diabetes.

## Electronic supplementary material

Below is the link to the electronic supplementary material.

ESM Table (PDF 132 kb)

## Abbreviations

AHEI: Alternative Healthy Eating Index
A:P: Animal protein-to-potassium ratio
FFQ: Food frequency questionnaire
HPFS: Health Professionals’ Follow-up Study
MET: Metabolic equivalent task
NEAP: Net endogenous acid production
NHS: Nurses’ Health Study
NHS2: Nurses’ Health Study II
PRAL: Potential renal acid load
