# Supplementary material for: Diet-dependent acid load and type 2 diabetes: pooled results from three prospective cohort studies
Source: Diabetologia. 2016 Nov 17;60(2):270–9. doi: 10.1007/s00125-016-4153-7 (PMC5831375; doi:10.1007/s00125-016-4153-7)
Supplement: Supplementary file 1 — (PDF 132 kb) [file 125_2016_4153_MOESM1_ESM.pdf]

ESM Table 1: Pooled hazard ratios (95%CI) of type 2 diabetes for dietary net endogenous acid production (NEAP) by strata of BMI, smoking status, hypertension, kidney stones and age.

	Quintile 1	Quintile 2	Quintile 3	Quintile 4	Quintile 5	P-trend	P-interaction
**By BMI**[b]							0.4237
BMI < 25 kg/m	1.00	1.05	1.09	1.14	1.33	<0.0001	
		(0.86, 1.29)	(0.87, 1.37)	(1.05, 1.24)[a]	(1.13, 1.57)[a]		
Overweight	1.00	0.99	1.05	1.06	1.24	<0.0001	
		(0.84, 1.18)	(0.91, 1.22)	(0.96, 1.18)	(1.12, 1.38)[a]		
Obesity	1.00	0.98	1.00	1.06	1.14		
		(0.88, 1.09)	(0.85, 1.19)	(0.88, 1.27)	(0.96, 1.35)	0.020	
**By Smoking**[c]							0.3272
Never smokers	1.00	1.00	1.04	1.09	1.23	<0.0001	
		(0.85, 1.18)	(0.86, 1.27)	(0.92, 1.28)	(1.10, 1.39)[a]		
Ever smokers	1.00	1.05	1.09	1.13	1.20	<0.0001	
		(0.96, 1.16)	(0.96, 1.24)	(1.02, 1.25)[a]	(1.08, 1.33)[a]		
**By Hypertension**[d]							0.8875
No hypertension	1.00	1.03	1.17	1.13	1.25	<0.0001	
		(0.88, 1.20)	(1.07, 1.27) [a]	(1.04, 1.23)[a]	(1.15, 1.36)[a]		
Hypertension	1.00	1.03	1.03	1.12	1.17	0.0356	
		(0.92, 1.14)	(0.81, 1.30)	(0.92, 1.35)	(0.99, 1.38)		
**By Kidney stones**[e]							
No kidney stones	1.00	1.02	1.05	1.06	0.99		0.3089
		(0.80, 1.30)	(0.84, 1.31)	(0.78, 1.44)	(0.70, 1.25)	0.5701	
Kidney stones	1.00	1.03	1.07	1.11	1.11		
		(0.91, 1.15)	(0.91, 1.20)	(0.99, 1.25)	(0.99, 1.29)	0.3551	
**By Age**[e]							
<60 years	1.00	1.01	1.07	1.07	1.19		
		(0.85, 1.19)	(0.87, 1.31)	(0.89, 1.28)	(1.02, 1.38)	<0.0002	
≥ 60 years	1.00	0.99	1.03	1.09	1.13		NA[f]
		(0.84, 1.18)	(0.87, 1.22)	(0.87, 1.37)	(0.98, 1.31)	0.0070	

Pooled results from NHS, NHS2 and HPFS using random-effect meta-analysis. [a]*P*<0.05; [b]adjusted for total energy intake (quintiles), family history of diabetes (yes/no), menopausal status (pre or postmenopausal (never, past or current menopausal hormone use), presence of hypertension and hypercholesterolemia (yes/no), smoking status (never smoker, former smoker, current smoker: 1-14, 15-24 or ≥ 25 cigarettes/d), alcohol drinking (g/d: 0, 0.1-4.9, 5.0-14.9, 15.0-19.9, 20.0-29.9, and ≥30), moderate/vigorous intensity activities (hours/week: 0, 0.01-1.0, 1.0-3.5, 3.5-6.0, ≥6), glycemic load (quintiles), AHEI-index (quintiles; including the following components: sugar containing beverages, fruit, vegetables, nuts, red meat, whole grains, EPA, DHA, other PUFAs, transfat, and sodium) and Western diet. [c]adjusted for total energy intake (quintiles), family history of diabetes (yes/no), menopausal status (pre or postmenopausal (never, past or current menopausal hormone use), presence of hypertension and hypercholesterolemia (yes/no), alcohol drinking (g/d: 0,

0.1-4.9, 5.0-14.9, 15.0-19.9, 20.0-29.9, and ≥30), moderate/vigorous intensity activities (hours/week: 0, 0.01-1.0, 1.0-3.5, 3.5-6.0, ≥6), body mass index (continuous), glycemic load (quintiles), AHEI-index (quintiles; including the following components: sugar containing beverages, fruit, vegetables, nuts, red meat, whole grains, EPA, DHA, other PUFAs, transfat, and sodium) and Western diet. [d]adjusted for total energy intake (quintiles), family history of diabetes (yes/no), menopausal status (pre or postmenopausal (never, past or current menopausal hormone use), presence of hypercholesterolemia (yes/no), smoking status (never smoker, former smoker, current smoker: 1-14, 15-24 or ≥ 25 cigarettes/d), alcohol drinking (g/d: 0, 0.1-4.9, 5.0-14.9, 15.0-19.9, 20.0-29.9, and ≥30), moderate/vigorous intensity activities (hours/week: 0, 0.01-1.0, 1.0-3.5, 3.5-6.0, ≥6), body mass index (continuous) glycemic load (quintiles), AHEI-index (quintiles; including the following components: sugar containing beverages, fruit, vegetables, nuts, red meat, whole grains, EPA, DHA, other PUFAs, transfat, and sodium) and Western diet. [e]adjusted for total energy intake (quintiles), family history of diabetes (yes/no), menopausal status (pre or postmenopausal (never, past or current menopausal hormone use), presence of hypertension and hypercholesterolemia (yes/no), smoking status (never smoker, former smoker, current smoker: 1-14, 15-24 or ≥ 25 cigarettes/d), alcohol drinking (g/d: 0, 0.1-4.9, 5.0-14.9, 15.0-19.9, 20.0-29.9, and ≥30), moderate/vigorous intensity activities (hours/week: 0, 0.01-1.0, 1.0-3.5, 3.5-6.0, ≥6), body mass index (continuous), glycemic load (quintiles), AHEI-index (quintiles; including the following components: sugar containing beverages, fruit, vegetables, nuts, red meat, whole grains, EPA, DHA, other PUFAs, transfat, and sodium) and Western diet. [f] Due to age-stratification in cox proportional hazard model
The results were pooled using random-effect meta-analysis.
